# Subcutaneous versus intravenous administration of Trastuzumab: a minimization cost analysis with real world data from a reference cancer centre in Peru

**DOI:** 10.3332/ecancer.2024.1708

**Published:** 2024-05-31

**Authors:** Iris Otoya, Natalia Valdiviezo, Katia Roque, Zaida Morante, Tatiana Vidaurre, Silvia P. Neciosup, Mónica J. Calderón, Henry L. Gomez

**Affiliations:** 1 Departamento de Medicina Oncológica, Instituto Nacional de Enfermedades Neoplásicas, Lima 15038, Perú; 2 Post Graduation Programme in Medicine, UNINOVE, Sao Paulo 03155-000, Brasil; 3 Institute of Investigations in Biomedical Sciences (INICIB), Universidad Ricardo Palma, Lima 15039, Perú; 4 Oncosalud, Auna Ideas, Lima 15036, Perú; a https://orcid.org/0000-0001-6185-9853; b https://orcid.org/0000-0002-4300-5958; c https://orcid.org/0000-0003-3267-7107; d https://orcid.org/0000-0003-2952-6068; e https://orcid.org/0000-0003-1995-4560; f https://orcid.org/0000-0002-2657-9853; g https://orcid.org/0000-0003-4935-7927; h https://orcid.org/0000-0003-2660-1843

**Keywords:** breast cancer, HER2 positive, Trastuzumab, subcutaneous, intravenous, cost, Peru

## Abstract

Breast cancer (BC) is a global concern, with Peru experiencing a high incidence and mortality. Trastuzumab, a crucial treatment for human epidermal growth factor receptor 2-positive BC, is administered intravenously or subcutaneously (SC). This study evaluates the costs associated with both methods at Peru's Instituto Nacional de Enfermedades Neoplásicas. Real data indicate that SC administration reduces treatment costs by approximately S/15,049.09. Cross-continental comparisons highlight a global trend favouring SC administration for efficiency and cost-effectiveness. The analysis provides insights for informed decision-making in resource-constrained healthcare settings like Peru, emphasising the need to consider local contexts in optimising oncology care.

## Background

According to GLOBOCAN 2020, breast cancer (BC) diagnosis has been increasing worldwide. It ranks as the fifth leading cause of overall cancer death, but for women, BC has the highest incidence and mortality rate, with a frequency of 24.5% and 15.5%, respectively [1]. In most Latin American countries, this pattern repeats [2]. Although in Peru, stomach cancer has the highest mortality in women, BC still prevails as the most diagnosed with an incidence of 18.5% in the whole country and resulting in up to 1,825 deaths in 2020 [1]. Moreover, in Metropolitan Lima alone new cases from 2013 to 2015 accounted for 18.2% of all malignant neoplasms and 12.9% of female deaths [1, 3].

For this type of cancer, its standard practice consists of evaluating the human epidermal growth factor receptor 2 (HER2) protein’s status. HER2 is found expressed in the membrane of some cancer cells and is involved in promoting cell growth and survival; therefore, it is crucial in determining the most effective therapeutic strategy [4]. HER2 overexpression is present in approximately 15%–20% of malignant breast neoplasms and is characterised by its aggressiveness with a high recurrence rate and fairly short disease-free survival (DFS) time after adjuvant treatment [4, 5].

Trastuzumab is a humanised monoclonal antibody that, by homodimerisation, inhibits the HER2 signaling pathway that promotes growth [6]. This drug has been approved by the FDA since 1998 for the treatment of HER2-positive metastatic BC as monotherapy or in combination with chemotherapy [6, 7]. The synergistic effect of Trastuzumab with that of chemotherapy, i.e., taxanes paclitaxel and docetaxel, reduces the risk of recurrence and mortality by half, in contrast to a regimen composed by chemotherapy alone; resulting in a 10% of absolute improvement in long-term DFS and a 9% increase in 10-year overall survival [8]. Trastuzumab is also approved in patients with node-positive disease and in N0 patients with tumours >1 cm, due to the relatively high risk of relapse; in addition, it is effective for both early stage and metastatic BC [8, 9].

Trastuzumab is administered via intravenous (IV) infusion every 3 weeks with a dosage calculated according to the patient's weight [10] or employing a subcutaneous (SC) fixed-dosage formulation administered by a single-use injection device [11]. Different studies have shown that subcutaneous Trastuzumab (SC-TZM) can offer many advantages compared to intravenous Trastuzumab (IV-TZM) and that it is effective in the treatment of patients with early stage HER2-positive BC, according to the primary endpoints of pathologic complete response (absence of invasive neoplastic cells in the breast, remaining ductal carcinoma *in situ*) [10, 12, 13]. Likewise, there is no significant difference between the two formulations with respect to adverse effects and event-free survival rates up to 3 years after completion of treatment [12, 13].

This study’s purpose is to evaluate the costs related to the administration of intravenous and SC-TZM at a public healthcare centre in Peru. To accomplish this, we considered the time invested by our staff in preparing and administering the drug. Regarding costs, we calculated the cost of Trastuzumab, materials for drug preparation, supplies needed for administration, infusion chair use and personnel involved in the process.

## Methods

The study was done in the Instituto Nacional de Enfermedades Neoplásicas (INEN), Lima–Peru; a national reference centre for the care of oncology patients.

We created a simple spreadsheet model using Microsoft Excel 2000 (Microsoft Corporation, USA) to evaluate the application costs per dosage of Trastuzumab (SC and IV presentation). All the costs used in our calculations were taken from the price list of the Department of Pharmacy of the INEN [14] based on the 2019 rate and expressed in the national currency, soles (S/.). INEN uses standardised prices for the only IV and SC trastuzumab presentations available at the said institution.

The study is focused on the healthcare payer perspective, since the costs are intended to be paid by the Seguro Integral de Salud (SIS), a Peruvian health insurance aimed at residents who do not have any other insurance. The findings will be relevant for decision-making entities that are responsible for allocating resources and funding healthcare interventions.

The time horizon for this study refers to the period of treatment (1 year) with each type of Trastuzumab administration, from the first dose to the last one (18 cycles total). The costs of treating adverse events were not included in the analysis.

To properly evaluate the cost and time of each administration, we divided it into the next elements.

### The drug

The nomenclature of SC-TZM used is 120 mg/mL/5 mL in vial form. The nomenclature of IV-TZM used is 21 mg/mL/20 mL, this specific drug was a biosimilar used in vial form. A single vial of Trastuzumab has a different cost depending on the type of administration (SC-TZM: S/. 4,212, IV-TZM: S/. 4,348) [14].

The treatment protocol for SC presentation was 18 fixed doses of 600 mg with a 3-week interval between doses. SC-TZM required a single vial for each administration. For IV-TZM presentation, it was an initial dosage of 8 mg/kg of body weight followed by 17 maintenance dosages of 6 mg/kg of body weight with a 3-week interval between doses. Calculations were made considering a mean of 27.54 BMI from a previous study with patients from INEN [15] We used two vials for the first dosage and one vial for the remaining 17 doses.

All treatments were administered according to the INEN's Care and Application of Specialised Medical Oncology Treatment Protocols.

### Materials for drug preparation

Cost of any material involved in the preparation of SC-TZM and IV-TZM. Calculations were made for a single dose (1 cycle) and total treatment (18 cycles).

### Supplies for drug administration

Cost of any supply used in the administration of SC-TZM and IV-TZM. Calculations were made for a single dose and total treatment.

### Infusion chair use

The cost of Specialised Medical Oncology Treatment Application on an outpatient by SC-TZM and IV-TZM are obtained from the Nursing and Chemotherapy Procedures tariffs from INEN. Calculations were made for a single dose and total treatment.

### Personnel involved in the process

The personnel involved consisted of a pharmaceutical chemist for drug preparation and a licensed nurse specialist in Oncology for drug administration. There is a doctor scheduled for supervision in each treatment room but since they do not have a specific time designed for each patient and the monitoring is made to the whole room at once, time and cost of patient supervision were not considered in this analysis.

### Drug preparation

Both drugs were stored at 2°C–8°C, following FDA recommendations and previous studies [11, 16, 17]. Since there was no difference in storage, this was not included in analysis.

Average preparation time did not include the time for transferring supplies from the warehouse to the pharmacy, the pre-labeling of the input material and the time for dressing change of the operators; only the time it took for the pharmaceutical chemist to reconstitute the IV-TZM presentation. Preparation cost was calculated using the salary per minute of a pharmaceutical chemist

### Drug administration

Administration time is defined as the time from the patient sitting in the infusion chair to the moment in which they get out of the chair at the end of each treatment cycle. Administration cost was calculated using the salary per minute of a licensed nurse specialist in Oncology.

### Statistical analysis

We conducted a descriptive analysis in RStudio. Packages such as ‘gtsummary’ and ‘tidyverse’ were installed to obtain tables. No other analysis were made since at the time of analysis when SC-TZM was available, there was only one provider for both SC and IV-TZM at INEN. Therefore, there was a single, fixed cost for each form of drug administration at INEN’s Department of Pharmacy.

### Ethical considerations

The study was approved by the Ethics Review Board of the INEN (22-0278) and conducted in compliance with all relevant ethical guidelines.

## Results

Considering the prices of the Department of Pharmacy of the INEN [14] and that IV presentation depends on body weight, as mentioned in Methods, we used the cost of two vials for the first dose of IV-TZM, S/. 8,696, and one vial for each subsequent administration, S/4,348. Given the 18 cycles of complete treatment, we have a total cost of IV-TZM being S/.73,916. On the other hand, SC-TZM costs S/.4,212 each vial and offers a fixed dosage of 600 mg, freeing the protocol from variations associated with weight. This characteristic offers greater predictability in medical expenses, avoiding the possible economic fluctuations that IV-TZM would present. SC-TZM gives a total of S/.75,816 while IV-TZM amounts to S/.82,612 (Table 1). There is a saving of S/.6,796 in favour of SC-TZM.

If we evaluate the cost assigned to materials for drug preparation for each method, we can see a substantial saving in consumables in the SC presentation, S/. 1.15, in comparison to S/.22.22 for IV-TZM in a single dose. The main difference is the price of the pump infusion line without volutrol (Table 2). The total cost for this category regarding SC-TZM is S/.20.70, meanwhile in IV-TZM is S/.399.96 (Table 3). The saving equals S/.379.26 in favour of SC-TZM.

Likewise, the cost of supplies needed for drug administration for a single-dose application is 32 times more expensive for IV-TZM than SC-TZM (S/.60.8 versus S/.1.89, respectively, Table 4), and considering the 18 cycles, this translates to a saving of S/ 1,060.38 (S/.1,094.40 versus S/.34.02, respectively, Table 5). The biggest difference in cost is due to the Dehp-Free pump infusion line for cytostatics, bifurcated extension of two needle-less valves, and sodium chloride 900 mg/100 mL (Table 4).

The cost of a Specialised Medical Oncology Treatment Application on an outpatient by SC-TZM is S/. 1.00 and by IV-TZM is S/. 39.00. In general, the cost related to the use of an armchair for SC presentation represents a saving of approximately 97% (S/.18 versus S/.702) in 18 treatment cycles compared to IV administration (Table 6).

The cost of Pharmacy Staff time, from the Central Mixing Area, and Nurse Time associated with treatment preparation was estimated by dividing the average monthly salary by the 150 hours scheduled in 1 month to calculate the hourly cost, giving a salary of S/. 23.33 and S/. 2.57 per hour and minute, respectively (Table 7).

Each vial for IV-TZM application is reconstituted with 7.2 mL of sterile water per injectable preparation for a single dose containing 21 mg/mL of Trastuzumab. One should not shake the product as excessive foaming during reconstitution could cause problems with the amount of Trastuzumab that can be extracted from the vial. If this occurs, the vial must be left resting for approximately 5 minutes [18]. Considering those recommendations, we have a total preparation time of 15 minutes for each IV-TZM application. Meanwhile, SC-TZM does not need any preparation time. Then, the time for SC-TZM is 270 minutes less in total (Table 8). The Preparation cost is S/.38.55 per cycle for IV-TZM, giving a saving of S/. 639.90 using the calculation of the salary per minute of a pharmaceutical chemist (Table 9).

The drug administration time is defined as the time from the patient sitting in the infusion chair to the moment in which they get out of the chair at the end of each treatment cycle. For IV presentation, the starting dosage should be administered as an IV infusion over 90 minutes. Patients are observed for 6 hours since the start of the first infusion and for 2 hours since the start of subsequent infusions for symptoms such as fever and chills or other infusion-related symptoms. If the starting dosage is well tolerated, subsequent doses may be administered as a 30-minute infusion [18]. For SC presentation, the recommended dosage is 600 mg administered by SC injection (under the skin) over 2–5 minutes. Monitoring is needed in case of adverse effects during administration and for at least 30 minutes after the first administration and for 15 minutes after subsequent administrations [18].

Then, SC-TZM drug administration time lasts 35 hours and 15 minutes less than IV-TZM (4 hours and 45 minutes versus 40 hours, Table 10). The cost associated with these times, using the calculation of the salary per minute of a licensed nurse, is S/. 732.45 for SC-TZM and S/.6 168.0 for IV-TZM (Table 11), giving a saving of S/. 5,435.55.

In summary, we spent S/. 76,621.17 in the whole treatment with SC-TZM and S/. 91,670.26 in IV-TZM (Table 12), with a total difference of S/. 15,049.09 which equals a saving of 16.42%.

## Discussion

Approved initially by all major regulatory agencies for the treatment of HER2+ metastatic BC, trastuzumab was expanded in 2006 for the treatment of early-stage HER2-positive BC [6, 9]. Adding trastuzumab to chemotherapy has improved the prognosis of this population. The evidence shows a significant reduction in recurrence (RR 0.66, 95% CI 0.62–0.71; *p* < 0.0001) and death (RR 0.67, 95% CI 0.61–0.73; *p* < 0.0001) compared to chemotherapy alone [9, 19, 20]. Furthermore, the demonstrated clinical effectiveness and safety of the medication reinforces its significance in managing early-stage BC [21, 22].

Biosimilar drugs, with comparable efficacy and safety to original biologics but lower production costs, are being increasingly integrated into clinical practice [23]. In HER2+ early BC, trastuzumab biosimilars have shown similar outcomes to the original product [24, 21]. Despite their potential to improve cancer care affordability, biosimilar uptake in Latin America, including Peru, has been slow due to various barriers such as regulations, legislature and market opportunities [25]. Currently, Peru has registered only two trastuzumab biosimilars of intravenous presentation since 2019 [14, 26]. Even though biosimilars represent lower spending costs, SC-TZM may offer potential time and cost-associated advantages, that are particularly important in resource-limited settings such as our country, where SC-TZM was only available for a short time during 2019.

Effective management of limited resources is critical to ensuring treatment access in public facilities, highlighting the critical role of economic evaluation in trastuzumab uptake and access in INEN, Peru. The limited public funding provided by SIS requires well-informed managerial decisions based on the costs associated with different ways of administering drugs [27, 28]. This evaluation aims to inform public management decisions and to add to the existing literature on the costs of oncology treatment, which can influence decisions on allocating resources in the health care system.

During our study, we examined real data to compare the expenses of administering 18 cycles of intravenous versus SC-TZM to patients with adjuvant HER2-positive BC. Our findings indicate that SC application reduces total treatment costs by approximately S/15,049.09, approximately 4,071.72 USD. A previous study in Peru, which used a simulation for cost analysis, estimated a direct cost reduction of S/.246.812,20 for the treatment of 100 patients with SC-TZM or S/.2,468.11 per patient [28]. It should be recognised that simulations might not capture all the variability and complexity of the real system, which may limit the extrapolation of results. However, the actual data reflect current conditions. The convergence of the results suggests that there is a consistent trend toward the economic advantages of the SC route of administration. In addition, it was observed that patient weight has a significant impact on the dosing of IV-TZM, as 80% of the patients weighed over 55 kg and thus required two doses of IV-TZM. This finding is of great importance in the Peruvian context where, according to the report ‘Perú: Enfermedades no transmisibles y transmisibles – 2021,’ 36.9% of the adult population is overweight and the average weight of women is 57 kg [29, 30]. The weight-based dosing of IV-TZM, which means that IV-modality may be comparatively more expensive in Peru, increases the cost.

The administration time of an oncology treatment is significantly influenced by the choice between subcutaneous and IV-TZM. Administration of SC-TZM reduces the administration time by 330 minutes because the drug is available in a ready-to-use formulation, eliminating the drug preparation process and subsequently reducing the use of clinical resources such as chemotherapy chairs and the time of healthcare personnel. Similarly, a previous Peruvian study found that, although the SC-TZM resulted in a longer post-injection monitoring time, the time spent by nursing staff was reduced due to the lack of infusion equipment, indicating a benefit for the SC-TZM. This pooled analysis highlights the significance of including direct administration times as well as operational and monitoring aspects when assessing the benefits of various modalities for administering trastuzumab [28].

Similarly, Lopez-Vivanco *et al* [31], calculated the healthcare professional’s time (HCP) as the mean sum of task times for all IV and SC administrations, the mean patient infusion chair time and treatment room time. The transition from IV to SC trastuzumab reduced active HCP time by half (27.2 minutes versus 13.2 minutes per cycle) due to the lack of IV catheter use, line flushing and drug reconstitution. A 78%–85% reduction in chair time and a 59%–81% reduction in patient’s treatment room time with SC-TZM resulted in 24 hours of free time for the 18 cycles. The respective saving was €979.60 for one complete treatment cycle [31].

Based on McCloskey *et al* [32] systematic review, we conducted a cross-continental comparison to determine similarities and differences in the trends of administering SC and IV Trastuzumab. This comparison aims to understand how global discoveries are reflected in different regions and how these might be contextualised in the Peruvian setting, thus providing a more comprehensive perspective [32].

In Europe, studies have shown SC administration to be more efficient and less expensive. For example, in Germany, trastuzumab SC administration has a significantly shorter total time of 5.4 minutes compared to 30 minutes for the IV formulation [33]. In Italy, when evaluating three different scenarios: IV-TZM, SC-TZM and IV-TZM followed by SC-TZM, they found that SC-TZM saved the most time. The reduction was 71.7% and 89.3% in preparation time and chair time use, respectively, compared to IV-TZM. However, total mean costs were not significant (IV-TZM: €14,233; SC-TZM: €14,272; and IV-TZM+SC-TZM: €14,535; *p* = 0.959), SC administration still proved to be a valuable option [34].

The results indicate a significant reduction in both time and cost, which suggests a positive bias toward implementing the SC formulation. When compared to data from Oceania, specifically New Zealand, a similar trend was observed, showing a reduction in both chair time and total nursing time. This resulted in a financial benefit of $76.94 (in New Zealand dollars) per patient per cycle [35].

A study conducted in Chile included direct and indirect medical costs associated with the preparation and administration of Trastuzumab (adjusted for body weight) and also costs due to serious adverse drug reactions and non-medical costs that occurred during the course of the 18 cycles. Results were primarily due to the number of vials per body weight [36]. Given the tendency for overweight in our patients, the use of SC-TZM instead of IV-TZM may have a significant economic impact on our public health system. Likewise, Brazil, Mexico and Panama have also shown reductions in total treatment costs and preparation and administration time with the use of SC administration [32, 37, 38]. These results confirm the operational and economic benefits of using the SC formulation, showing a comparable pattern to our results and those observed in other regions.

This comparative enhances comprehension regarding how the advantages of the subcutaneous formulation of trastuzumab are mirrored across various healthcare systems, and how these results can guide clinical and policy determinations in managing HER2-positive BC. However, there was variability in data among various research and medical entities, emphasizing the necessity of taking into account the local context, clinical protocols and evaluation methods in interpreting outcomes and devising SC therapy implementation strategies.

In addition, there is a preference for SC-TZM versus IV-TZM administration in patients with HER2-positive early BC. In 2013, 124 patients were randomised to receive SC-TZM followed by IV-TZM, and 124 to receive the reverse sequence. SC-TZM via the single-use injection device was preferred by 216 patients (*p* < 0.0001). Also, the reported adverse events occurred in 58% of patients during the combined SC periods and in 44% of patients during the combined IV periods; in which only 3% and 2%, respectively, were grade 3, mostly influenza; with no grade 4 or 5 events [39]. Therefore, patient preference makes an important point to consider at the moment of deciding on treatment, since not only reduces costs and improves time use, but also the patient’s disposition and quality of life.

## Conclusion

The decision to deliver Trastuzumab SC or IV requires a comprehensive evaluation of time efficiency, cost, clinical need, patient preference and a framework that promotes optimal oncologic care and resource allocation. This analysis provides valuable information to inform current clinical practice and to inform future research aimed at optimising oncology care.

The results obtained within the study showed that the use of SC-TZM, compared to the IV version, generates less costs associated with an overall saving of 16.42% (18 cycles), which means less time spent in the preparation and application of the treatment, as well as a reduction in patient’s chair time. These time reductions translate into financial savings, more efficient use of resources and improved quality of care.

## Conflicts of interest

The authors declare that they have no conflict of interest.

## Funding

This study is self-funded. The authors declare they have no financial or personal relationships with other organisations that could inappropriately influence their work.

## Author contributions

All authors conceived and designed the study. All authors contributed to the article and approved the final version of the manuscript.

## Figures and Tables

**Table 1.** Cost of drug by presentation (expressed in local currency: Soles).

	Subcutaneous (120 mg/mL/5 mL)	Intravenous (21 mg/mL/20 mL)
Cost of vial	S/. 4,212	S/. 4,348
First administration	DOSAGE: 600 mg	DOSAGE: 8 mg/kg
	S/. 4,212	S/. 8,696
Subsequent administration (17 cycles)	DOSAGE: 600 mg	MAINTENANCE DOSAGE: 6 mg/kg
	S/. 71,604	S/. 73,916
TOTAL (18 cycles)	S/. 75,816	S/. 82,612

**Table 2.** Cost of materials for drug preparation for a single dose (expressed in local currency: Soles).

	Subcutaneous		Intravenous	
	Quantity	Price (S/.)	Quantity	Price (S/.)
Disposable hypodermic needle N°18 G × 1 ½ in	1	0.11	1	0.11
Pump infusion line without volutrol	0	0	1	21.07
Disposable syringe 20 mL with needle 21 G × 1 ½ in	1	0.54	1	0.54
Sterile surgical gloves N°7	1	0.54	1	0.54
TOTAL		1.15		22.22

**Table 3.** Total cost of materials for drug preparation (expressed in local currency: Soles).

	Subcutaneous	Intravenous
First administration (1 cycle)	S/ 1.15	S/ 22.22
Subsequent administration (17 cycles)	S/ 19.55	S/ 377.74
TOTAL (18 cycles)	S/ 20.7	S/ 399.96

**Table 4.** Cost of supplies for drug administration for a single-dose (expressed in local currency: Soles).

	Subcutaneous		Intravenous	
	Quantity	Price (S/.)	Quantity	Price (S/.)
Paracetamol 500 mg tab	4	0.32	4	0.32
Chlorphenamine Maleate 10 mg/mL Iny 1 mL	0		1	0.13
Ranitidin 25 mg/mL Iny 2 mL	0		1	0.54
Sodium chloride 900 mg/100 mL Iny 100 mL	0		3	9.09
Dehp-free pump infusion line for cytostatics	0		1	23.43
Disposable hypodermic needle Nº 18 G × 1 1/2 in	0		2	0.22
Disposable syringe 20 mL with Needle 21 G × 1 1/2 in	2	1.08	2	1.08
Disposable syringe 5 mL with Needle 21 G × 1 1/2 in	0		1	0.11
Peripheral intravenous catheter Nº 24 G × 3/4 in	0		2	2.48
Antiseptic wipe with chlorhexidine gluconate 2% and isopropyl alcohol 70%	1	0.49	1	0.49
Adhesive transparent dressing 6 × 7 cm	0		1	1.84
Bifurcated extension two Needle-less valves × 12 cm	0		1	21.07
TOTAL		1.89		60.8

**Table 5.** Total cost of supplies for drug administration (expressed in local currency: Soles).

	Subcutaneous	Intravenous
First administration (1 cycle)	S/ 1.89	S/ 60.8
Subsequent administration (17 cycles)	S/ 32.13	S/ 1,033.6
Total (18 cycles)	S/ 34.02	S/ 1,094.4

**Table 6.** Cost of chair use (expressed in local currency: Soles).

	Subcutaneous	Intravenous
First administration (1 cycle)	S/ 1.00	S/ 39.00
Subsequent administration (17 cycles)	S/ 17.00	S/ 663.00
Total (18 cycles)	S/ 18.00	S/ 702.00

**Table 7.** Cost of personnel involved (expressed in national currency: Soles).

	COSTS		
	**Monthly salary**	**Salary per hour**	**Salary per minute**
Pharmaceutical Chemist (Central Mixing Area)	S/. 3,500.00	S/. 23.33	S/. 2.57
Licensed Nurse Specialist in Oncology (Infusionist)	S/. 3,500.00	S/. 23.33	S/. 2.57

**Table 8.** Total time of drug preparation.

	Subcutaneous	Intravenous
First administration (1 cycle)	0 minutes	15 minutes
Subsequent administration (17 cycles)	0 minutes	255 minutes
Total (18 cycles)	0 minutes	270 minutes

**Table 9.** Total cost of drug preparation time (expressed in national currency: Soles).

	Subcutaneous	Intravenous
First administration (1 cycle)	-	S/ 38.55
Subsequent administration (17 cycles)	-	S/ 655.35
Total (18 cycles)	-	S/ 693.9

**Table 10.** Total time of drug administration.

	Subcutaneous	Intravenous
First administration (1 cycle)	30 minutes	360 minutes (6 horas)
Subsequent administration (17 cycles)^a^	255 minutes (4 horas, 15 minutes)	2,040 minutes (34 horas)
Total (18 cycles)	285 minutes (4 horas, 45 minutes)	2,400 minutes (40 horas)

a Time spent in subsequent administrations (per cycle): 15 minutes SC versus 120 minutes IV

**Table 11.** Total cost of drug administration.

	Subcutaneous	Intravenous
First administration (1 cycle)	S/. 77.1	S/. 925.2
Subsequent administration (17 cycles)^a^	S/. 655.35	S/. 5242.8
Total (18 cycles)	S/. 732.45	S/. 6,168.0

a Cost of in subsequent administrations (per application): S/.38.55 SC versus S/.308.4 IV

**Table 12.** Final cost of treatment by presentation.

	Subcutaneous	Intravenous
Cost of drug	S/. 75,816	S/. 82,612
Cost of materials for drug preparation	S/. 20.70	S/. 399.96
Cost of supplies for drug administration	S/. 34.02	S/. 1,094.40
Cost of chair use	S/. 18.00	S/. 702.00
Cost of drug preparation time (pharmaceutical chemist)	-	S/. 693.90
Cost of drug administration (nurse)	S/. 732.45	S/. 6,168.00
TOTAL	S/. 76,621.17	S/. 91,670.26
